# Mechanisms of action and processes of yoga-based group intervention for inpatients with schizophrenia spectrum disorders–A longitudinal qualitative study

**DOI:** 10.3389/fpsyt.2023.1086468

**Published:** 2023-02-07

**Authors:** Laura Töbelmann, Inge Hahne, Theresa Schulze, Niklas Bergmann, Lukas Fuchs, Marco Zierhut, Eric Hahn, Kerem Böge

**Affiliations:** Department of Psychiatry and Psychotherapy, Charité Campus Benjamin Franklin, Charité – Universitätsmedizin Berlin, Freie Universität Berlin, Humboldt-Universität zu Berlin, Berlin Institute of Health, Berlin, Germany

**Keywords:** yoga, mindfulness, schizophrenia spectrum and other psychotic disorder, longitudinal qualitative study, mind-body medicine

## Abstract

**Background:**

Research exploring the effects of yoga therapy (YT) on individuals with schizophrenia spectrum disorders (SSD) is scarce. Therefore, the current study aimed to explore possible mechanisms of actions and processes, as well as adverse effects of a novel yoga-based group intervention (YoGI) for in-patients with SSD in a German university hospital setting.

**Material and methods:**

A longitudinal qualitative study was integrated into a rater-blinded randomized controlled trial, exploring the impact of a 4-week YoGI as add-on treatment. In-depth interviews were conducted with participants receiving YoGI (*n* = 19) in addition to treatment as usual (TAU) and a control group (*n* = 14) which only received TAU. Interviews were conducted at baseline (*n* = 33) and 4 weeks post-intervention (*N* = 28) to assess the participant’s experiences and how they changed over time. The interviews (*N* = 61) were audio-taped, translated, coded, and analyzed by means of inductive thematic analysis. Separate case summaries were prepared for each participant to analyze longitudinal changes within subjects. The research team members collaboratively discussed the final list of themes and subcodes. Rater-based questionnaires, such as the Positive and Negative Syndrome Scale (PANSS), Calgary Depression Scale for Schizophrenia (CDSS), and Personal and Social Performance Scale (PSP) were administered at baseline to assess clinical outcomes.

**Results:**

At baseline, participants reported a desire to improve their stress- and symptom management. A minority of participants expressed reservations toward yoga, and several psychosocial barriers were named, including worries about symptom exacerbation. At post-intervention, four mechanisms of change became evident from the interviews: (1) acquiring competence in relaxation, (2) increased interoceptive awareness, (3) feeling connected, and (4) a sense of spiritual wellbeing. A small number of participants reported difficulties with YoGI.

**Conclusion:**

Generally, YoGI positively influenced participants’ experiences of their inpatient stay, regarding distress, self- and body awareness, social connectedness, and spiritual wellbeing. However, participants also illuminated necessary adjustments to improve the intervention. YoGI will therefore be adapted and further developed in an iterative process based on a participant involvement approach. The efficacy regarding outcomes and processes needs to be investigated in a future larger-scaled randomized controlled trial.

## 1. Introduction

Schizophrenia-Spectrum-Disorders (SSD) are a group of complex and heterogenous primary psychotic disorders that affect about 2.6% of the adult population in Germany (1). Altogether, SSD core symptoms can be differentiated between positive symptoms (e.g., hallucinatory experiences, delusional beliefs), negative symptoms (e.g., lack of motivation, affective flattening, alogia, and social withdrawal), and cognitive impairment (difficulties in attention, memory, and executive functioning) (2). SSD are frequently accompanied by comorbidities such as depression, anxiety (3, 4), and metabolic syndrome due to side effects of antipsychotic medication (5, 6). Approximately half of the affected individuals undergo extensive hospital-stays (7), with an average treatment duration between 21 and 32 days for in-patients with SSD in Germany (8). Furthermore, SSD is characterized by frequent relapses (9), with high rehospitalization rates of up to 40% within the first 2 years (10, 11). These impact the quality of life and psychological wellbeing of affected individuals and have long-term negative effects on their functioning in interpersonal relations and occupational interaction with co-worker (12, 13). Altogether, these factors contribute to SSD being associated with the highest treatment costs per patient of all medical diseases (14–16). The first line of treatment for SSD, according to international (NICE) and national guidelines (DGPPN-S3), includes pharmacological agents combined with psychological treatments, such as cognitive behavioral therapy (CBT) (17, 18).

Several studies highlight the efficacy of antipsychotic medication in the treatment of positive symptoms, but show less improvement of negative and cognitive symptoms, as well as the quality of life (19–22), which highlights the relevance for cost-effective adjunctive inpatient therapies. In recent years, a growing body of evidence suggests the therapeutic effectiveness of non-pharmacological interventions for individuals with SSD (23, 24). In this context, there is an increasing emphasis on mindfulness-based interventions, including Yoga Therapy (YT), as an adjunctive treatment that could promote both physical and mental facets of wellbeing (25–29). The term “Yoga” is derived from the Sanskrit origin “*yui*” meaning “*union*” referring to the union of mind, body, and spirit (30). It is an ancient spiritual and philosophical discipline, that originated in northern India and aimed to promote human health. While there is no definite taxonomy of Yoga, common elements of YT incorporate exercises including physical postures (Asanas), breathing exercises (Pranayama), meditation techniques, and components of deep relaxation (31). It involves mild to moderate physical activity and is therefore suitable for patients with low exercise tolerance (32, 33). Various meta-analyses report amelioration of clinical symptoms with YT, demonstrating small-to-moderate effects for positive (34, 35), negative (34–37), and depressive symptoms (35, 38–40) in individuals with SSD. Furthermore, several studies show improvements in cognitive deficits in speed indices of attention (41–43), as well as improved working memory (43) in outpatients with SSD following YT versus physical exercise or waitlist control group. Additional studies highlight improvements in facial-emotion recognition (44, 45), and socio-occupational functioning (45–47). A recent meta-analysis (48) showed not only large effect sizes for MBIs and small effect sizes for YT, but also highlighted that no serious adverse events were reported (*N* = 1,774 in 43 RCT). These results suggest that MBIs are well-accepted and safe interventions for individuals with SSD. Other studies have shown large effect sizes of YT on stress (49) and anxiety (49, 50), as well as increased psychological wellbeing (50) and large effect size on quality-of-life improvement (32, 51, 52) following YT versus usual care. These positive effects remained stable over a period of up to 6 months follow-up (53). Stress and anxiety are often related to the worsening of psychotic symptoms in acutely ill patients with SSD (54). Therefore, developing a repertoire of stress-reducing coping strategies is an important component of early therapeutic support (55, 56). Another important source of distress for individuals with SSD is the high prevalence of internalized stigma, shame, and low levels of self-compassion (57–59), which is further associated with a worse prognosis (58). Recent studies emphasized that individuals with SSD who learn to develop a mindful attitude experience less self-stigma and distressing thoughts related to their symptoms (60, 61). These effects may be directly linked to specific coping strategies provided for individuals with SSD, that are not otherwise addressed by standard therapeutic options (27). Such strategies include bringing non-judgmental awareness to the present moment (62) and reducing mental engagement from internal and external stressors, including those attributed to psychotic experiences (26, 29).

Overall, most RCTs on YT for patients with SSD were conducted with outpatient samples (41, 42, 44, 46, 55, 63–65). Furthermore, the quality of available research on YT for individuals with SSD displays heterogeneous results and often lacks methodological rigor, due to missing reported outcomes, small sample sizes, and a wide variety of employed yoga styles (53, 66). As shown in a Cochrane Review published in 2017, low availability of high-quality data favoring YT does not allow a clear recommendation into the guidelines of treatment (67), illustrating the relevance of future studies in inpatient settings.

The mechanisms of action in YT are still largely unknown. Before further assessment of the effectiveness of YT for SSD, it is important to improve the understanding of patients’ experiences with these interventions. Two previous qualitative studies among patients with SSD suggest that YT induces relaxation and stress-reduction, which lead to improved focus (68), an experience of interconnectedness, increased self-compassion, and effects on symptom representation (69). These findings align with quantitative research results.

Yoga-based Group Intervention (YoGI) is specifically developed for the treatment of in-patients with SSD, and is based on previous qualitative studies conducted by the research team, focusing on the general structure of MBIs (70) and the specific conditions under which YT can succeed (69). The present study is a further study and features a qualitative longitudinal research design (LQR) for the first time. This study aims to decipher the mechanisms behind the effect of the interventions and to understand how and why YT leads to perceived changes in symptom presentation. The study examines (1) the initial attitudes of participants toward Yoga, aiming to gain deeper understanding of individual barriers and facilitating factors to predict the (un)responsiveness to YT and (2) behavioral changes, mechanisms of action, as well as desired and adverse effects. This in-depth approach is an invaluable tool for improving our understanding of participants’ experiences over time, which are less clearly depicted in quantitative or cross-sectional data alone (71). A comprehensive perspective is expected to evaluate the impact of the intervention and can highlight necessary treatment adaptations. The findings aim to support further improvement of future yoga interventions for SSD in-patients by supporting further participative and iterative development of an evidence-based yoga manual tailored to this patient group.

## 2. Materials and methods

### 2.1. Design

**FIGURE 1:**
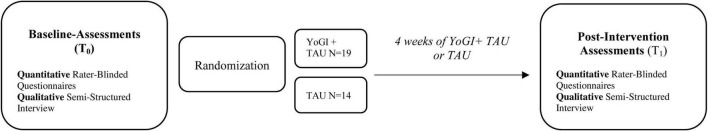
Study design.

A qualitative longitudinal study was integrated into a monocenter rater blinded RCT (Hahne et al. in process; preregistered at clinicaltrials.gov: NCT04730518), comprising YoGI as an add-on treatment to the regular inpatient treatment as usual (TAU). Participants included in the study were randomly allocated to two groups: the control group received TAU, while the experimental group received YoGI in addition to TAU. Rater-blinded assessments, as well as qualitative semi-structured interviews were completed at baseline (T_0_) and at post-intervention (T_1_) 4-weeks later (see Figure 1). Participants who received TAU were interviewed as well, at both time points (T_0_ and T_1_). This allows for a more differentiated comprehension of potential mechanisms at action and possible impacts of YoGI adjunct to the comprehensive standard treatment. Data collection took place between May 2021 and June 2022.

### 2.2. Recruitment

Recruitment of participants took place on the ward for psychotic disorders at Charité –Universitätsmedizin Berlin, Campus Benjamin Franklin. All inpatients were screened and the eligibility to part-take in the study was assessed by a multidisciplinary team in weekly medical rounds. After being informed about the study by the study assistants and expressing willingness to participate, patients were verified according to the following *inclusion criteria*: (a) diagnosed with a Schizophrenia-Spectrum-Disorder (SSD) (ICD-10: F20–F29) by a qualified psychiatrist, (b) aged between 18 and 75 years, (c) able to communicate effectively in German language, and (d) able to give written consent about their willingness to participate in the study. *Exclusion criteria* were defined as (a) any neurological disorder, (b) any item rated > 6 on the P-Scale of the PANSS suggesting an acute psychosis with severe psychotic symptoms, (c) acute substance use or substance abuse (except nicotine) according to ICD-10 criteria assessed by a qualified psychiatrist, (d) acute suicidality assessed by a qualified psychiatrist, and (e) contemporaneous co-therapies such as electroconvulsive- or ketamine therapy.

### 2.3. Participants characteristics

In total, *N* = 33 participants (*n* = 19 YoGI+TAU and *n* = 14 TAU) from the ward for psychotic disorders at the Charité – Universitätsmedizin Berlin were included in the present study. Of these participants, *n* = 20 participants were diagnosed with schizophrenia (F20), *n* = 6 with schizoaffective disorder (F25), *n* = 4 with acute polymorphic psychotic disorder (F23), *n* = 2 with delusional disorder (F22), and *n* = 1 with schizotypal personality disorder (F21), all according to ICD-10 criteria. Except for one person (*n* = 1), all received psychotropic medication. There were no significant differences between both conditions on any demographic and clinical measures at baseline, which is displayed in detail in Table 1. There was a total of *n* = 5 dropouts (*n* = 2 YoGI+TAU and *n* = 3 TAU), all due to premature discharge against medical advice. Therefore, no post-intervention (T_1_) interviews could be conducted with these participants.

**TABLE 1:** Semi-structured interview guide.

**Baseline interview (T_0_)**
1. What is concerning you at the moment? Could you tell me why you have been admitted to the hospital? 2. What are your goals and wishes for your inpatient treatment? 3. How motivated do you feel to participate in the psychotherapeutic offers on the ward? 4. How would you determine a successful therapy? 5. Do you have prior experience with yoga or other mindfulness techniques such as meditation?
**Post-intervention interview (T_1_)**
1. How have you been feeling in the last few days? 2. Could you tell me about your last couple of weeks? 3. Four weeks ago, you presented with (x, y, z)–what about these symptoms now? 4. How has the treatment here supported you in realizing your goals? 5. Which of the therapies did you find particularly helpful? • What did you experience during the therapeutic intervention? • What did you perceive regarding your symptoms during and after the therapeutic intervention? • What did you manage well and what did you enjoy in this therapeutic intervention? • Did you experience any difficulties during the therapeutic interventions? If so, of what type were they? • Do you have any suggestions which could improve the intervention? • Is there anything that you have learned in this therapeutic intervention that you would like to take with you into your future life? 6. Were there any therapies that did not help you?

### 2.4. Treatment as usual

Treatment as usual (TAU) consisted of the comprehensive standard care inpatient treatment offered at the ward for SSD at the Charité – Universitätsmedizin Berlin. An interdisciplinary team with multiple professionals such as psychiatrists, clinical psychologists, social workers, occupational therapists, physiotherapists, and psychiatric nurses was carrying out the interventions. Each patient received an individualized weekly therapy plan according to official treatment guidelines. The treatment program included pharmacological treatment, individual cognitive behavioral therapy (CBT) once a week and several group therapies such as concentration practice, psychoeducational groups, metacognitive training, self-confidence training, occupational therapy, physiotherapy, and physical exercise.

### 2.5. Yoga-based group intervention

The 50-min class, as an add-on to TAU, was held weekly by a clinical psychologist and a yoga teacher as a co-therapist, who helped with mental support and hands-on-alignments during the intervention. The class was openly offered to all patients in the in-patient ward, regardless of study participation. Group sizes varied between 3 and 10 participants. YoGI was tailored specifically to patients with SSD. The intervention was based on a semi-structured manual (Table 2). The manual was developed continuously in close collaboration with various psychologists, yoga teachers, as well as the patient’s. The criteria for the asanas selected were that these would be doable for all participants regardless of their physical fitness. The structure of YoGI was supported by preliminary findings of our research team (69, 70). The results of the studies highlight the importance of adaptations to deal with potential challenges and ensure inclusivity. The yoga mats were arranged in a circle so that all participants faced each other as well as the teacher. This was chosen to create a heightened sense of security and to support a group feeling. Props such as blocks, and blankets were provided to assist the performance of the postures. Participants with physical limitations could participate from a chair and were given separate instructions in this case. Following recommendations concerning implementing mindfulness for psychosis, prolonged phases of silence were kept to a minimum and basic anchoring methods were used, as well as comprehensible language (72, 73). Instructions emphasized curiosity about bodily sensations and encouraged participants to find a balance between “ease” and “effort” in relation to their physical engagement in the practice. Therefore, choices to modify postures, to stay in or return to a particular posture, were always offered. YoGI started with an invitation to bring awareness to what is present in that moment. Afterward, there was room for patients to share their intentions or wishes for the upcoming class. The participants were guided through a sequence that included posture sitting, standing, and laying down. The class ended with a deep relaxation *savasana*, where participants were guided through a brief body scan or listened to relaxing music. Afterward, there was a final round, where they could share about their experience.

**TABLE 2:** Sociodemographic variables for both conditions at baseline.

Variable	YoGI + TAU (Y)*	TAU (T)*	*X*^2^/t (df)	*p*
	*n/mean (SD)*	*n/mean (SD)*		
Sex			0.65 (1)	0.421
*Male*	7	8		
*Female*	12	6		
Age	*44.26 (15.23)*	*48.07 (15.34)*	0.68 (31)	0.5
Nationality			0.21 (1)	0.648
*German*	16	10		
*Other*	3	4		
ICD-10 diagnosis			6.39 (5)	0.27
*F20*	10	10		
*F21*	–	1		
*F22*	2	–		
*F23*	4	–		
*F25*	3	3		
Length of current hospital stay (weeks)	*8.58 (4.29)*	*9 (6)*	0.023 (31)	0.822
Duration of illness in years	*11.63 (12.45)*	*18.85 (12.15)*	1.60 (31)	0.119
Highest educational achievement			4.34 (4)	0.362
*Lower secondary school*	3	3		
*Higher secondary school*	3	3		
*A-Level*	5	5		
*Vocational training*	3	–		
*University degree*	5	3		
Occupation			1.76 (3)	0.624
*Unemployed*	7	5		
*In retirement*	6	6		
*Student*	2	–		
*Employed*	4	3		
Previous experience with yoga			0.19 (1)	0.665
*Yes*	8	4		
*No*	11	10		
PANSS				
*Positive Syndrome Scale*	*18.47 (4.49)*	*20.79 (4.3)*	1.43 (31)	0.161
*Negative Syndrome Scale*	*21.21 (61)*	*24.14 (5.89)*	1.40 (31)	0.172
PSP	*57.79 (13.21)*	*53.5 (12.98)*	−0.89 (31)	0.378
CDSSS	8.21 (4.41)	8.5 (4.22)	0.18 (31)	0.856

*P*-values are based on Chi-square test for categorical and *t*-tests for continuous variables. YoGI, yoga-based group intervention; TAU, treatment as usual; SD, standard deviation; PANSS, Positive and Negative Syndrome Scale; PSP, Personal and Social Performance Scale; CDSS, Calgary Depression Scale for Schizophrenia.

*Quotes from participants of YoGI start with “Y” + number and from TAU with “T” + number.

Italic values represent the statistical variables.

Participants’ attendance was documented at each yoga class. All (*n* = 19) YoGI + TAU participants attended 4/4 yoga sessions.

### 2.6. Quantitative measures

Rater-blinded assessments were conducted by a resident physician of psychiatric medicine or a licensed psychologist at baseline (T_0_). Assessments included three rater-based questionnaires to assess the severity of various clinical symptom dimensions.

#### 2.6.1. Positive and negative symptoms

Psychotic symptoms were assessed through the Positive and Negative Syndrome Scale (PANSS) (74). PANSS is a widely established clinical interview that examines positive and negative symptoms. In the current study, two scales (Positive Scale and Negative Scale) were used, with each subscale consisting of 7 items. Each item is accompanied by a definition as well as anchoring criteria for all rating points, which stand for increasing levels of psychopathology (1 = absent; 7 = extreme) (74). The PANSS displays a good internal consistency, good interrater reliability, and construct validity (75, 76).

#### 2.6.2. Depression

The Calgary Depression Scale for Schizophrenia (CDSS) (77) aims to assess the level of depression independently from negative symptoms in individuals with schizophrenia. The scale contains 9 items, and each item has a four-point anchored measure (0 = absent; 3 = severe). Item 1–8 are questions for a clinical interview to measure depression, hopelessness, self-deprecation, guilty ideas, pathological guilt, morning depression, early wakening, and suicidal ideation to assess the presence of symptoms over the past 2 weeks. The last item is based on the interviewer’s observations during the assessment. A cutoff point of eight is considered an indicator of comorbid depression. The CDSS has good internal reliability, inter-rater-reliability, and discriminant and convergent validity (78, 79).

#### 2.6.3. Social functioning

The Personal and Social Performance Scale (PSP) (80) is a measurement tool to assess the domains of psychosocial performance in personal, social, and occupational functioning. Four areas are assessed: (1) socially useful activities including work and study, (2) personal and social relationships, (3) self-care, and (4) disturbing and aggressive behavior. It contains a final global score rating of 1–100; a higher score represents a higher level of personal and social function. The stable test-retest reliability (ICC = 0.79) and good validity and inter-rater reliability validity (81–83) of PSP suggest that the scale is a valid measure of overall social functioning (84).

### 2.7. Interviews

The present study collected qualitative data through 61 interviews (YoGI *n* = 36, TAU *n* = 25). The interview language was German. A semi-structured interview guideline with open-ended questions was prepared, based on two previous qualitative studies of the research team (69, 70). The interview guide (see Table 3) was created through an iterative process by the researchers involved in this study, including a consultant psychiatrist, a psychotherapist, clinical psychologists, and a sociologist, to assess the content and ensure comprehensible wording. Patient participation was invited during a pilot phase consisting of five interviews. After each interview, feedback on the guideline interview questions was obtained and integrated. All interviews were conducted face-to-face by one interviewer LT, a white, female-identified medical student (6th year) who is a certified yoga teacher and co-instructed the YoGI sessions with IH over a period of 4 months. LT received interview training by KB and LF. The interviews were audio recorded. Questions were asked openly and a detailed response to the question was encouraged.

**TABLE 3:** Procedure of yoga-based group intervention (YoGI).

Components	
**• Duration:** 50 min **• Set-up:** Mats/chairs arranged in a circle **• Props:** blocks, blankets **• Instructions:** encourage optionality using keywords like *“allow,” “notice*,” and invitational phrases like “*if you like*,” *“if it feels good*,” *“when you are ready”*	
**Procedure**	**Duration**
**Seated mindful body scan** and set intention for the practice	5 min
Seated postures*	10 min
- Sukhasana (Easy Pose) - Seated Side Stretch - Seated Spinal Twist - Bitilasana Marjaryasana (Cat and Cow Stretch) - Utthita Chakravakasana (Sunbird)	
**Standing postures:** *Modifications instructed for Participants on chair*	20 min
- Surya Namaskar (Half Sun Salutation) - Virabadhrasana 2 (Warrior 2) - Utthanasana (Standing Forward Bend) - Utthita Trikonasana: (Extended Triangle Pose) - Prasarita Padottanasana (Wide legged Standing Forward Bend) - Vrikshasana (Tree Pose)	
**Seated postures**	
- Navasana (Boat Pose) - Ardha Matsyendrasana (Half Lord of the Fishes Pose)	
**Restorative postures:** *Modifications instructed for Participants on chair*	5 min
- Setu Bandha Sarvangasana (Shoulder Bridge) - Ananda Balasana (Happy Baby Pose) - Jathara Parivartanasana (Reclined Spinal Twist)	
**Deep relaxion:** guided mindful body scan	5 min
- Savasana (Supine Rest)	
**Final round:** sharing about participants experiences	5 min

*Annotation: these postures are named in Sanskrit language, as listed in this book (172).

The baseline interview (T_0_) comprised a list of five open-ended questions and lasted between 6 and 39 min (on average 19 min). The post-intervention interview (T_1_) contained 12 open-ended questions and took between 6 and 61 min (on average 32 min). The 6-min interviews were conducted with a patient with catatonic symptoms, which complicated her engagement in the conversation. Questions in the baseline interview (T_0_) centered around the current mental state of the participants, wishes and goals for the inpatient stay, their motivation regarding the psychotherapeutic treatment options and their prior experience with yoga and mindfulness. The post-intervention interview (T_1_) included questions regarding the participants’ overall experience with the psychotherapeutic treatment options, discussing which therapies were found to be particularly helpful and which therapies were not experienced as supportive. Moreover, it was explored what experiences were made during the therapeutic intervention and what was learned during the intervention if regarding a transfer into daily life. Questions emphasized the overall experience of the in- patient stay and participants were asked to talk about their three most favorable therapies. This way of phrasing was intended to minimize the implication that participants were supposed to speak specifically about YoGI, reducing social desirability bias. In light of expressed concerns about a “positivity bias” in mindfulness research (85) (due to researchers’ sympathy for the mindfulness practice and their interest in promoting it), the research design was compiled beforehand to reduce and actively reflect these proclivities (86). In accordance with COREQ guidelines (87) the authors acknowledge the unique relationship between the researcher and the participants, which is especially sensitive in longitudinal qualitative research (88). Participants and researcher LT have been in contact before and after the interview on the ward for psychotic disorders solely for study appointments for YoGI.

### 2.8. Data safety

To ensure data confidentiality according to “Datenschutz Grundverordnung” (DGSVO) data privacy law, all data was pseudonymized. All electronic data was password protected and stored on secure data servers hosted by the Charité – Universitätsmedizin Berlin. Electronic tablets were used administer quantitative data using the software REDCap which works with encoded connections and manages data in the case reports. Audio recorders and tablets were stored in lockers to which only selected members of the research team had access. The pseudonymous data will be stored for a maximum period of 10 years at Charité – Universitätsmedizin Berlin.

### 2.9. Data analysis

Data was analyzed following inductive Thematic Analysis (89), a well-established method for reporting and analyzing data and describing patterns in detail. As LQR is “not tied to any particular methodology or analytical approach” (88), thematic analysis was chosen, because it is suitable to give voice to the individual’s subjective experience in a longitudinal qualitative study design (88, 90) while offering a structured mode of categorizing the data. Throughout the complete analysis, the researchers engaged in a process of continuous critical reflection on their own preconceived notions and ideas. Therefore, they paid particular attention to the fact that themes do not naturally reside in the data but instead reflect on the active, interpretative, and selective role of researchers within the analytical process (89).

When a case number of 30 participants (*n* = 50 interviews) was reached, the data analysis began. 11 additional interviews were collected until code and meaning saturation set in (91). From this point onward, data collection and data analysis occurred simultaneously as it is commonly practiced in LQR (92). Data was systematically managed using MAXQDA20, a qualitative analysis software to systematically manage and store data. The following process were conducted:

1. The audio-taped interview data was verbatim transcribed by LT.
2. All transcripts (YoGI + TAU and TAU; T_0_ and T_1_) were thoroughly read in random order by LT and TS to familiarize themselves with the data (93). Afterward, the first coding round focused on identifying relevant chunks of data regarding yoga and sorting them into broad codes. The second round of coding was done to identify sub-codes related to yoga within previously highlighted data. In line with inductive techniques, the views of participants were prioritized over *a priori* theoretical constructs.
3. In the third round of coding, LT identified patterns among the codes and established a full list of themes, subthemes, and codes.
4. The next step was to analyze longitudinal experiences with YoGI within subjects. For this purpose, T_0_ and T_1_ interviews of one person were read consecutively. A separate case summary was prepared for each YoGI participant by LT and TS. The case summaries were based on the predefined themes and subthemes previously defined in the third round of coding. The analysis here moved from descriptive to exploratory and aimed to uncover the mechanisms behind the impact of the intervention and the causes of change or lack of change over time. LT and TS engaged in a constant and close exchange to ensure the validity of the analysis.
5. Several research team members (LT, TS, IH, NB, KB, and LF) then met to build consensus and clarify the final list of themes and subcodes. Analytical descriptions were produced for each code, including name, definition, and anchor examples.
6. In the final phase, illustrative quotations were chosen and translated into English language by LT. The quotes selected were representative of the participants, with special attention to divergent opinions. Lastly, the analytical report was produced.

### 2.10. Ethical considerations

The study was approved by the local ethical committee of Charité – Universitätsmedizin Berlin, Germany (EA4/239/20) and preregistered at clinicaltrials.gov: NCT04730518. The object of the study was fully explained to the participants and written consent about participation and audiotaping was obtained before the interview. The interviewer LT emphasized the optionality of taking part in the study. Furthermore, the participants were informed of their right to withdraw from the study and stop the interview at any time. Participants were not financially compensated for their participation.

## 3. Results

**FIGURE 2:**
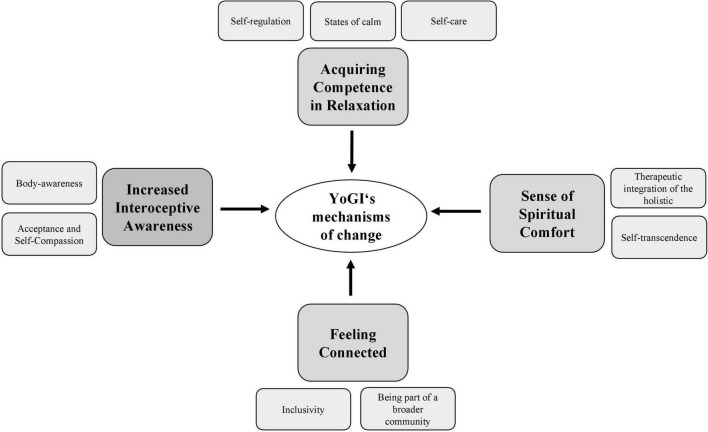
Yoga-based group intervention (YoGI’s) mechanisms of change.

The qualitative findings begin with a review of the results at baseline, which address *motivators*, and *barriers* related to yoga practice. The second part of the results presents four psychosocial mechanisms of action attributed to YoGI (Figure 2 and Table 4).

**TABLE 4:** Mechanisms, subthemes, codes, and quotes.

Mechanisms of change	Subthemes	Codes	Illustrative quote
1. Acquiring competence in relaxation	States of calm	• Feeling balanced, grounded, and centered • Importance of deep relaxation (Savasana)	“So, in the yoga group it was very relaxing for me. I was also a bit jittery again before […]. I have to say it really calmed me down (Y7).” “The relaxation and tranquility that you have with yoga. […] mentally and physically, you could feel it. One was really relaxed (Y5).”
	Self-regulation	• Detaching from thoughts • Guiding attention to somatic sensations • Inserting control by conscious breathing • Moving mindfully to distract from internal stressors	“For example, when I lie awake at night, I try to synchronize my breathing with this sound (acoasms) which is there (Y13).” “I think that was this moment when we were supposed to close our eyes, imagine something, that’s when your whole body just relaxed, and you got rid of all your thoughts (Y10).” “I then try, even if I’m not doing so well, that I then actively relax […] I just also tried to do myself a little bit of good and hold myself. I try to feel into myself, how I sit and to sit in such a way that I feel well. If I feel bad, I will also sometimes take the position of the child (Y18).” “The images in my head that I have from time to time: after yoga, it brought the stress level down like that, […] after that there was really a little bit less images, a little bit less stress, so always at least one step further down and that’s actually a lot (Y16).”
	Self-care	• Integrating self-soothing actions • Taking breaks	“I need that, I’ve noticed. Maybe I’ll take these breaks in between more and I’ll pay more attention to breathing (Y15).”
2. Increased interoceptive awareness	Body-awareness	• Experience of body-mind-connection • Perceiving and acknowledging needs, limits, impulses	“I also notice I have better body awareness (Y12).” “I’ve also been doing yoga this morning and there’s always relaxation and that always does my body good too and body and mind belong together like that (Y5).” “Well, what was a little bit difficult for me is that I’m a little bit heavier and some things were difficult, like holding arms and stuff, you have to give up or feel your limits before, but I actually coped well with that (Y18).”
	Self-compassion	• Building capacities for introspection • Reduction of performance orientation • Reduction of body-shame	“Because that’s not about winning or being better, it’s about agility and being balanced and that’s what I love about it (Y9).” “At the beginning I was always so tense and thought “oh and hm” and then I represented myself again and that I was fine and told the yoga teacher that I was in pain and that we had an agreement that if it got too bad, I would stop. And that I had that option, I thought that was really good and that’s why the last few times I was then able to experience that in a really relaxed way (Y9).”
	Difficulties with introspection	• Resistance to get in touch with inner experience • Fear of losing control	“(In Savasana), I was afraid that I would be hypnotized […] that I would no longer have my own will (Y2).” “There where you had to breathe like that or do something with your eyes closed, that was uncomfortable for me (Y11).”
3. Feeling connected	Inclusivity	• Everyone can participate • Support and connection • Cultivating compassion for others	“On TV, yes, they always just showed the positions that you, that I couldn’t have done, but first of all, you’re doing this for beginners, and you can participate as much as you can (Y17).” “So, it was always a very peaceful and social situation (Y18).” “Because the people who have never done yoga, they’re included there, so it doesn’t even feel like you can’t participate in it (Y13).”
	Being part of a community	• Being a yoga practitioner • Yoga practice as a social reference point	“Surprisingly, it wasn’t frustration with yoga. Normally I get annoyed and frustrated relatively quickly, but not with yoga, because I thought to myself: No, I want to do this for myself, I want to be able to do this! And then I was more motivated (Y16).”
4. Sense of spiritual comfort	Self-transcendence	• Experiences of letting go, protection, inner peace • Turning toward the soul	“(That feeling after yoga), I know that from the swimming pool, but this was a little different feeling because here was body and soul at the same time (Y14).” “Then I thought to myself: that’s exactly it. You must let go! and that was somehow really great (Y9).” “When I feel meditative, I feel that I relax, that I also feel protected and that my head is free (Y12)”
	Therapeutic integration of the holistic	• Yoga as healing modality • Soothing existential dread • Desire for holistic therapeutic approaches	“If I didn’t have that (yoga) […], I would already be in intensive care or something. Because it still gives me such strength to bear everything (Y13).” “I also had 1–2 yoga classes at (another) clinic when I was an inpatient there and that was just insanely sporty, but the holistic is then just missing and I find that much better solved here (Y18).”

### 3.1. Encountering yoga

#### 3.1.1. Motivators

**FIGURE 3:**
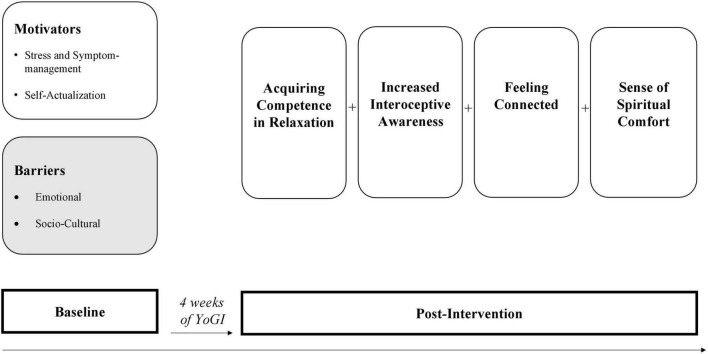
Longitudinal experience of yoga-based group intervention (YoGI).

Before the intervention period started, participants were asked about their prior experiences and attitudes toward yoga, to investigate facilitating factors and barriers to participation in YoGI (see Table 5 and Figure 3). Participants intention to engage in yoga, regardless of prior experience, was characterized by quiet optimism. Participants agreed that yoga was “*good”* (T10) and “*beneficial* (Y19),” or an “*unimaginable but interesting* (T12)” experience. Most of them hoped yoga would reduce their distress as well as strengthen control over their health status. Some of the participants also described an interest in self-transformation and “*personality development* (T8).” These ideas were based either on positive prior experiences with yoga and mindfulness or on reports from acquaintances and the media.

**TABLE 5:** Motivators and barriers reported at baseline (T_0_).

Themes	Subthemes	Codes
Motivators	Stress and symptom management	• Improvement of symptoms (pain, sleep disturbances, anxiety, delusional symptoms) • Relaxation • Learning self-regulation techniques • Physical fitness
	Self-actualization	• Personal growth • Self-transcendence/ spiritual aspiration
Barriers	Emotional	• No belief in health benefits • Fears of increased inner turmoil “monkey mind”
	Socio-cultural	• Perceived incompatibility with own belief system • Worries about exacerbation of (psychotic) symptoms

#### 3.1.2. Barriers

Some participants expressed skepticism about yoga. Shared among these participants was not having prior experience with yoga. An interviewee described not believing in the health benefits of yoga: “*I don’t think [yoga] will help now. It won’t at all (laughs). If I do a few exercises, I will not suddenly be healthy* (Y6).” Other participants also reported that they had difficulty with introspective practices in the past and experienced increased inner turmoil: *“So, practices where I close my eyes or have my body awareness, I can’t do with my eyes closed, it just makes me nervous* (Y16).” There were also reports of perceived incompatibility with one’s belief system because yoga was perceived as an “*esoteric* (T10*)”* practice. In addition, a participant reported: “*Sometimes I have such reservations about being introspective.”* The caution was justified by saying: “*I often refrained from it because I was afraid it might trigger psychosis, and it’s also generally said [by doctors] that meditation is contraindicated* (T8).” Some participants’ views of yoga became more positive throughout the intervention, thereby reducing negative preconceptions. Most participants stated that they wanted to continue practicing yoga after the intervention. Only four participants had no intention of continuing. Four mechanisms of change were identified based on participants narrations of how YoGI influenced their in-patient stay: (1) acquiring competence in relaxation, (2) increased interoceptive awareness, (3) feeling connected, and (4) a sense of spiritual comfort.

### 3.2. Acquiring competence in relaxation

#### 3.2.1. States of calm

Significant post-interventional change narrated by almost all participants was that they learned to *“relax”* and achieve a state of “*physical and mental ease* (Y2).” During and after YoGI, they described feeling “*really calmed down* (Y10)” and “*more balanced* (Y18)”: “*Well, that first [yoga] class I had such a relaxation that I hadn’t had in all the time before on the ward. […] So for the psyche it is very relaxing, very pleasant* (Y5).” The deep relaxation phase in the yoga class (*savasana*) was rated particularly positively by many participants: “*The final relaxation, that’s what I need the most* (Y5).”

#### 3.2.2. Self-regulation

Some participants further reported how they internalized self-regulatory techniques learned in YoGI and adopted them as habitual coping strategies for relaxation, dealing with anxiety, sleep disorders, distraction, and relieving pain. These strategies included detaching themselves from current experiences and thoughts by focusing attention on the body, and this was achieved through meditation or breathing techniques, such as deep abdominal breathing. Some participants also reported consciously assuming yoga postures (e.g., child’s pose) as coping strategies when feeling distressed. They concluded that the use of yoga techniques led to a reduction in internal turmoil. Consequently, these participants described a greater sense of control over their own physiology, as well as their symptoms: *“You make yourself aware of your feelings, and this awareness alone leads you to distance yourself a bit from your feelings so that they no longer have such a grip on you. I think that is an experience of self-efficacy that can really help yourself in certain situations* (Y4).”

#### 3.2.3. Self-care

Further, some participants also reported calmer states in their daily lives on the ward. They reported how the self-soothing learned in YoGI was incorporated into their daily activities. Participants gave themselves permission to “*take breaks* (Y19, Y16)” in moments of exhaustion or overwhelm. A participant reported recognizing the importance of taking “*time out from the stresses of everyday life*” and findin*g “peace in a free moment* (Y15)” for themselves.

### 3.3. Increased interoceptive awareness

#### 3.3.1. Body awareness

After the intervention, one of the most reported changes according to participants was an increased ability to notice bodily cues. Body-related mindfulness was found to be helpful in overcoming the feeling of disembodiment that is commonly experienced in patients with SSD (94, 95). This was also reported by participant in the control group (TAU). The participant explained that previous experiences with yoga were helpful to reconnect with the body: *“Exercises, stretches, breathing into the body and then just watching the breath, that’s good. Because now I also feel that I kind of lost my body, my body awareness* (T9).” It was described that YoGI is not an “*athletic-ambitious*” movement practice but creates space for the inner experience: *“For me the first yoga class is always particularly impressive because you completely switch over and listen to your body again […] that you get your body awareness back into consciousness. I then pay attention to that outside of the yoga group as well* (Y18).” Participants also highlighted a sense of togetherness and connection between mind and body. They described finding it easier to classify their bodily sensations. They could better differentiate interactions between physical sensations, thought, behavior, and feelings of stress: *“When I have strong feelings, on the one hand, I try to analyze them a little bit and on the other hand I try to manage them physically. […] Because the emotional experience then usually has physical consequences again, and if you address those, you can get away from it a bit* (Y18).”

#### 3.3.2. Self-compassion

At baseline, many participants exhibited a lack of self-confidence, fear of failure, and difficulty motivating themselves to practice yoga. Many saw YoGI as an opportunity for personal growth and self-awareness regarding these issues. In contrast, post-intervention participants described a greater acceptance of their actual states of being and self-compassion. In this regard, it was experienced as supportive that the instruction of yoga postures was combined with an active request to perceive one’s own needs and limits and perform exercises self-determinedly. Participants further described how YoGI enabled them to increase self-observation through the slow pace and exploratory stimuli in the teacher’s instructions. *“The yoga teacher always encouraged us to listen to ourselves and do something different if it made us feel better. That’s when I really practiced it. I’m someone who tends to try really hard to do everything perfectly in a class situation. So, it’s enriching for me to be able to step back and think more about myself* (Y18).” They also felt encouraged to distance themselves from high self-expectations and achievement-orientation. A participant reported that initially, the “*deficits*” (lack of flexibility and muscle strength) were the main focus, but as YoGI progressed, the attitude changed as well: *“From the second or third round, it became more comfortable, where I knew […] that I could consciously spare my body or take it back, so it wasn’t like I usually know: you must go faster, higher, further. That’s when I really came to rest* (Y16)*.”*

Furthermore, a new relationship to one’s own physicality was observed. Participants reported increased abilities to “*feel their (physical) boundaries* (Y9).” Notably, participants with severe obesity expressed how mindful movement served as a medium for self-kindness and acceptance, as they learned to relate differently toward their bodies. This led some participants to focus more on their physical capabilities instead of their limitations: *“It was exhausting, also because of my weight and so on […]. But it doesn’t matter, I still felt good that I was able to do it and that it just made me feel better and I felt more comfortable in my body* (Y7)*.”* Another report illustrates the possibility of perceiving and working on both, the body image and relationship to self: *“I told myself: yeah, I’m not as athletic as the others, but I’m participating, and that is worth a lot* (Y9)*.”*

#### 3.3.3. Difficulties with introspection

Some (*n* = 3) participants who were novel to yoga experienced adverse effects. They reported difficulties relating primarily to the introspective aspects of YoGI. This was due to the slow pace of the movements, as well as discomfort while closing their eyes. Two participants expressed skepticism about yoga practice at baseline because they did not believe in its efficacy or feared conflicts with their worldview. One participant reasoned how the deceleration and the invitation to introspection led to an increased awareness of the symptoms: *“The quiet also gave the symptoms more space and freedom again to come to me and bug me* (Y6)*.”* Two other participants concluded that the experience of entering a state of relaxation also increased fears of losing control. This was described as ruminating thoughts about *“losing one’s own will”* or being *“hypnotized* (Y2)” during the deep relaxation phase (*savasana*). Another participant reported: “*I felt like I was losing touch with reality. I felt like I was about to fall asleep or something […]. I think I’m just so used to being in a mild panic all the time. It’s been like that for the last 2 years and it feels like I don’t have that panic anymore. It is also pretty new* (Y11).”

### 3.4. Feeling connected

#### 3.4.1. Inclusivity

Taking part in a yoga class that is accessible to all, regardless of physical and mental difficulties, was repeatedly positively emphasized. It is notable, that this was reported by almost all participants, ranging from being physically fit and experienced with yoga to those with limitations. The experience of being able to participate despite mental and physical limitations led to a decrease in self-stigmatization and limiting beliefs described at baseline: *“This is actually a nice offer for everyone. Even if you’re disabled, you can still do certain techniques or exercises* (Y17)*.”* Sharing the experience of YoGI provided participants with a sense of belonging and mutual support. Participants reported that practicing together in a group was “*more enjoyable* (Y14)” than alone.

#### 3.4.2. Being part of a broader community

Participating in YoGI and despite many obstacles “*making it anyway* (Y7),” strengthened some participants’ sense of confidence. Participants were eager to identify as someone “who practices yoga.” Some reported doing yoga exercises with fellow patients outside of YoGI or arranging to meet friends after their hospital stay to practice yoga together. Therefore, practicing yoga in a group functioned as a new social reference point. In this way, processes of individual empowerment were embedded in the social dimensions of mental health (e.g., respect, connectedness, belonging), which was critical for participants, as it renewed aspects of their social identity.

### 3.5. Sense of spiritual comfort

#### 3.5.1. Self-transcendence

Many participants reported that yoga differed from other sports or exercises and related this to its introspective aspects. Participants described experiences of “*flow* (Y12, Y13, Y18),” “*inner peace* (Y10, Y16),” “*feeling at home in the body* (Y18),” “*letting go* (Y9),” having a “*free mind* (Y4),” and “*feeling protected* (Y12).” Many participants reported that yoga was different from other forms of sport or exercise and related this to its introspective aspects. For these participants, the yoga practice was an intimate process that included turning toward their “*soul* (Y12, Y14, Y15, Y9),” a term that was repeatedly mentioned in relation to the experience of YoGI. YoGI offered them a way to move beyond the here-and-now of psychiatric hospitalization: *“I thought it was beautiful: how can I explain it? That “turning inward,” you know you’re doing something for your body, and you’re so peaceful inside* (Y12).”

#### 3.5.2. Therapeutic integration of the holistic

Many (*n* = 13) participants from both conditions reported turning to yoga, meditation, and spirituality to cope with their illness and facilitate “*shifts in perspective* (T1).” For these participants, yoga and other mindfulness techniques were perceived as a path toward self-transformation and “*healing* (Y12, Y13, T1, T3, T9).” While some participants perceived it within the context of a secular stress-relief paradigm, others saw the yoga practice as a “*holistic* (Y18)” way of life through which they sought to achieve a sense of purpose and hope for their circumstances. Themes related to spirituality were brought up more often by participants who had prior experience with yoga or meditation. This was clearly illustrated by a participants report: *“If I didn’t have that (yoga) […], I would already be in intensive care or something. Because it still gives me such strength to bear everything* (Y13)*.”* Regular practice was a factor in the relationship between yoga and spirituality and appeared to be a key component of deepening yoga practice with experiences of inner peace and hope. Participants who were interested in spirituality perceived YoGI as a holistic therapeutic approach to managing and healing their illnesses. Yoga, as a movement practice embedded in a philosophical-spiritual doctrine, represented in this regard an interesting therapeutic option for these participants: *“I also had 1–2 yoga classes in (another clinic) when I was an inpatient there and that was just insanely sporty, but the holistic aspects were missing then, and I find that much better solved here* (Y18).”

These four psychosocial changes (acquiring competence in relaxation, increased interoceptive awareness, feeling connected and a sense of spiritual comfort) positively impacted participants’ overall wellbeing during their inpatient stay. Participants reported overall change by feeling more calm, confident, connected to others, empowered in their social identity, and using more flexible self-regulatory coping strategies to deal with symptom appraisal. For some participants, practicing yoga also led to a relief from existential fears and strengthened a sense of life meaning and purpose.

## 4. Discussion

The present study examined the longitudinal experiences of in-patients with SSD who took part in a novel YoGI for 4 weeks in a German university hospital setting. To the best of the authors’ knowledge, this is the first report of such a study worldwide. Prior to the intervention, participants reported various *motivators* as well as *emotional* and *sociocultural barriers* related to yoga practice, that influenced their participation as well as perception of YoGI. From this point of departure, the main analytical focus was on gaining novel insights into YoGI’s underlying processes and mechanisms. Four psychosocial mechanisms of action were identified based on participants’ experiences after taking part in YoGI: (1) *acquiring competence in relaxation*, (2) *increased interoceptive awareness*, (3) *feeling connected*, and (4) *a sense of spiritual wellbeing*. Participants reports shed light on how YoGI influenced their wellbeing, coping strategies, relationships with self and others, and spiritual experiences such as self-transcendence and hope. Furthermore, potential adverse experiences in relation to the yoga practice were explored.

### 4.1. Acquiring competence in relaxation

One mechanism of action is based on participants’ reports of reduced distress related to psychotic symptoms due to *acquiring competence in relaxation*–using various techniques they acquired during YoGI (e.g., breathing exercises, present-moment awareness, detachment, and mindfully moving into yoga postures). These findings are consistent with the qualitative literature on the stress-reducing effect of yoga for SSD (69, 96), as well as for other clinical populations (97–100), highlighting the transdiagnostic effects of yoga practice. The application of these self-regulatory techniques took place not only during YoGI. Participants reported using them spontaneously as flexible coping strategies when dealing with auditory hallucinations, anxiety, rumination, sleep disturbances, and pain during their inpatient stay. The enhanced ability to relax was perceived as a key benefit of YoGI, as the symptoms of SSD are related to and can be exacerbated by stress and anxiety (101, 102).

Furthermore, research suggests that it is not the occurrence of psychotic symptoms itself that leads to a distressing experience, but the individual’s reaction to these symptoms and the associated meaning (103, 104). Therefore, self-regulatory techniques may support more functional management of psychotic symptoms, through both acceptance of their presence and an active engagement with them, leading to desensitization and symptom decline through exposure (105–107).

### 4.2. Increased interoceptive awareness

Our qualitative findings on *increased interoceptive awareness* as a mechanism of action of YoGI, align with previous qualitative findings stating that yoga practice increases body awareness in non-clinical populations (108), as well as in individuals with schizophrenia (96). Participants of YoGI reported how increased interoceptive awareness and feeling more anchored within their body reduced anxiety, mirroring the mechanisms of MBIs overall in supporting individuals to acknowledge their anxiety. Increased interoceptive awareness also contributes to the ability to identify opportunities to take self-responsible action within the yoga practice (e.g., changing a pose if it causes physical pain or intensifying a stretch if it feels good). Here, a rediscovery of one’s ability to execute behavior occurred: the participants began to feel self-efficacious, and able to make decisions according to their own physical and emotional needs. A qualitative study conducted with adolescents with psychosis conceptualized personal empowerment as “taking control and making decisions for oneself” (109). These findings align with other studies indicating that self-determination and agency play a critical role in the recovery of schizophrenia, as it enables individuals to understand better their health-related needs (109–111).

Obesity is common amongst individuals with SSD (112–115), often leading to dissatisfaction with body image (116). Those study participants with obesity described a reduction of body-related shame, as well as increased self-compassion through experiences of positive embodiment in YoGI, such as being grounded and sensing the body vs. being in the head and focusing on physical deficits and limitations. These outcomes are in line with previous findings that show that yoga practitioners display a more positive body image than non-yoga practitioners (117) elucidating the effects of yoga practice on positive body image and increased self-acceptance as well as -compassion (118–122).

Alongside *increased interoceptive awareness*, participants in the present study stated how they gained meta-cognitive insights–defined by Flavell as “cognition about cognition” (123)–through connecting external actions (e.g., “I did the child’s pose”) to internal states (“I felt soothed”). Here, the bodily experience functioned as a vehicle for self-knowledge and self-regulation. These findings are in line with a recent systematic review (26) that proposed increased meta-cognitive awareness through mindfulness practice as an underlying mechanism of action for decreasing negative symptoms (124). Increased meta-cognition, as well as a furthered “knowledge about oneself” (125) and the capacity to distinguish oneself as separate from depressive and negative symptoms (e.g., negative thoughts), have shown to be effective in the treatment of SSD (126, 127)

In terms of a pluralistic psychiatric approach, YT could be an effective additional therapeutic option, as it promotes increased self-awareness and as self-efficacy. This could have a synergistic effect, increasing medication adherence, as impaired insight is one of the key drivers of medication non-adherence, contributing to impaired long-term functioning in SSD (128–130). Furthermore, research among patients with post-traumatic stress disorder has shown that yoga practice might promote engagement in psychotherapy (100, 131, 132), suggesting that yoga practice may increase awareness of internal states and strengthens emotional regulation, which occurs before effective engagement in psychotherapy (100, 133). Therefore, simultaneous participation in YT and psychotherapy could lead to better therapeutic outcomes in SSD as well as other psychiatric disorders than either modality alone, as they promote similar effects, such as emotion regulation and developing the capacity for introspection (132). Future studies might examine this relationship more closely by investigating the simultaneous engagement of both therapeutic modalities.

### 4.3. Feeling connected

Almost all participants considered the group format of YoGI to be valuable, although the format of YoGI included relatively little time devoted to verbal sharing. The results demonstrate the positive impact of group therapy–as a low-cost and low-threshold treatment modality–for individuals with SSD. The group format promoted *feeling connected* and receiving mutual support, temporarily easing social isolation and stigma (134, 135). Qualitative studies show that the relationship between instructor and participant, as well as between participants themselves, plays an important role in the successful delivery of MBIs (136). Positive social interaction has been empirically identified as an important recovery factor (137, 138). Participants in YoGI described the normalizing and empowering experience of being addressed as a “fellow human being” and being included for who they are, regardless of mental disorder and/or physical limitations. Previous literature highlights the importance of interpersonal recognition and acceptance for the recovery process in SSD (139–141). Nonetheless, participants continue to deal with social and mental illness-related stigma and exclusion outside of YoGI, impacting their treatment offers, identity and lifestyle. This touches on a limitation of YoGI, as it does not address the social context in which participants live. If limiting social conditions (e.g., high prices for yoga classes, low availability of inclusive, mental-health informed YT) are not considered, it is questionable how sustainable the therapeutic change can be in the long-term, as regular practice might be a key factor in the effectiveness of YT. This highlights the importance of integrating the social conditional aspects in the future development of YTs theory and practice for individuals with SSD.

### 4.4. Sense of spiritual wellbeing

The main findings within the mechanism of *spiritual wellbeing* were related to dimensions such as meaning in life, purpose, and hope. Spirituality is empirically linked to lower stress levels (142, 143) and studies suggest that it supports adaptive coping in individuals with mental disorders (144, 145). However, most of the yoga research, ignores the spiritual dimensions of yoga (146–149). Interestingly, despite the secular nature of YoGI, participants responses suggest that even a yoga practice without explicit spiritual teachings can influence aspects of spirituality. In line with previous studies (146, 150–152), the introspective character of the yoga class supported a sense of basic trust, openness, and helped to maintain hope in surviving life’s adversities, such as an acute psychotic episode. The distinction between YoGI and other therapies (such as exercise therapy) also becomes apparent through the analysis of the language used to describe participants experiences. These findings show that there is an emic difference between YoGI and other in-patient therapies, as participants used descriptions like “*connecting to the soul*” and “*inner peace*.” Participants narrations point to an important source of hope connected to YoGI: the simple moments in yoga class when participants transcended the here-and-now of their current psychiatric in-patient status and experienced moments of presence and flow. Exploring one’s spirituality may promote a person’s sense of hope for the future by rediscovering what is important to them and, therefore, may facilitate personal recovery (153–156). Participants reports imply that many turned to spirituality to cope with their illness and therefore welcome the therapeutic integration of holistic approaches. These findings are in line with a recent study that suggests the clinical incorporation of spiritual aspects to broaden the spectrum of evidence-based treatments for individuals with mental health conditions (157). The extent to which yoga, due to its comprehensive philosophy, is superior to other body-oriented methods and relaxation techniques (e.g., progressive muscle relaxation) should be further investigated, as empirical findings comparing effectiveness are rare (158).

### 4.5. Clinical implications for a safe yoga practice

Before and after the intervention, participants reported having the idea that yoga could help them to cope with their mental disorders, which motivated them to engage in YoGI. However, the provision of YT in an institutionalized context for patients with SSD is scarce. This is likely based on the myth that meditation can induce psychosis (159–162), causing skepticism and reluctance due to safety concerns. This view is held by both treatment providers and individuals with SSD themselves. In general, adverse events are insufficiently emphasized in both psychotherapy (163) and mindfulness research (85). A recent study about adverse events in relation to meditation practice pointed out that participants with pre-existing mental disorders were more likely to report unpleasant experiences as well as higher severity of those experiences, possibly due to strong identification with unpleasant emotions and a reluctancy to let them pass (164). Practicing yoga or meditation may lead to greater awareness of one’s physical sensations, feelings, and thoughts (165), and so subjecting individuals to an increased confrontation with their symptoms.

The current study shows that three out of 19 participants during YoGI experienced an increase in symptoms (e.g., ruminating thoughts, de-realization) or showed other adverse effects, such as anxiety about losing control during yoga practice (e.g., lying on the floor, closing their eyes). The qualitative nature of this study allowed an in-depth analysis of the difficulties arising during yoga practice. Thereby, the results of this study can inform about necessary treatment adaptations. Consistent with trauma-informed yoga protocols (166), strategies for dealing with these adverse phenomena include the yoga teacher’s a non-directive, empowering stance. This includes encouraging participants to modify the practice by offering different variations within the postures. For example, for the final relaxation (savasana), participants can choose to remain in a seated posture, instead of lying down on the floor with closed eyes. The awareness of having a choice can help participants to stay present in their experience and recognize that they are safe, even when uncomfortable sensations or feelings arise. Given the increased risk of adverse experiences, future yoga interventions for individuals with SSD should give more time for spontaneous expression of emotions and concerns so that strategies can be found to work collectively through the difficulties that may arise during practice. Participants of YoGI described didactic and structural aspects of YoGI that they perceived as supportive in regard to yoga practice (see Table 6). Yoga, as a practice that connects a person’s body, mind, and/or spiritual experience, could be a fruitful adjunct treatment for individuals with SSD, as it assists participants in accessing their personal resources. This is consistent with the paradigm shift in therapy for individuals with SSD: from a focus on disability to a focus on empowerment and recovery (155, 167–171).

**TABLE 6:** Aspects of didactics and rhetoric in yoga-based group intervention (YoGI) that were perceived as supportive by participants.

Theme	Codes	Illustrative quotes
Didactic aspects supporting relaxation	Fixed sequence and repetition Alternation between strenuous and relaxing postures Listening to calm music	“Yes. It [Savasana] was difficult at first, but if you do it more often, it worked (Y7).” “This interplay of sport and relaxation and rest, I think that’s the crucial thing. […] You get exerted, you also get a little sore muscle, but you can also relax again afterward, that’s the good thing about it (Y17).” “Also listening to the music during the meditations [Savasana], the melodies, that helps too (Y14).”
Yoga postures and breathing techniques	Child pose (closeness to the body, calming effect) Tree pose (challenging, rewarding, trains balance) Abdominal breathing (soothing, calming) Corps pose/Savasana (supports deep relaxation)	“So, putting hands on the mat in the child’s pose. So that helped reduce nervousness and anxiety (Y15).” “Take a deep breath, pay attention to abdominal breathing, and sometimes just stretch and let go (Y19).” “The final relaxation, that’s what I need the most (Y5).”
Rhetorical choices of the yoga teacher initiating mindful awareness	Attentive to the specific needs of participants Provide of exploratory stimuli and give various options to try out Provide guidance in making one’s own decisions Express permission for self-care and time-outs	“Everyone has been advised that if they have problems and don’t feel well, they can also come forward and have the opportunity to articulate themselves (Y13).” “After all, we were always encouraged by the yoga teacher to listen to ourselves and to do something different sometimes, if that makes us feel better. That’s when I really practiced it (Y18).”

### 4.6. Strengths and limitations

There are several important strengths and limitations to this study. To the best of the authors’ knowledge, this is the first qualitative longitudinal study evaluating persons with SSD experiences with yoga. Another strength of this study is the inclusion of a control condition, who received TAU, allowing a better distinction between the general effects of the in-patient stay and the specific mechanisms of action and processes of YoGI. Two researchers analyzed the data independently by two researchers to reduce the influence of subjective bias and to strengthen the results’ comprehensibility. Over 1 year of research, findings were regularly discussed within the research team, and thus improving intersubjectivity and the overall quality of the analysis through a stepwise analysis process.

There are also limitations, as qualitative data is inappropriate for determining the impact of the intervention on measurable mental health outcomes. We, therefore, conducted this qualitative study alongside a RCT in which quantitative outcomes of YoGI on multiple dimensions, including symptom representation and cognition, are assessed by displaying a mixed-methods design. This study aimed to investigate YoGI for in-patients who displayed residual symptoms, with chronic symptom representation (mean duration of illness for YoGI: 11.63 and TAU: 18.85 years) and moderate positive and negative symptoms, as well as comorbid depressive symptoms. For a limited period (4 months), the interviewer was involved in conducting YoGI, making the interviews more susceptible to social desirability bias. However, to minimize this effect, interviews did not explicitly inquire about YoGI, but rather asked about the participants’ three favorite therapies at the ward. An important limitation was the availability of a single coder due to cost and time constraints. Results are, however, based on a pretest-posttest design. Therefore, no conclusion can be drawn about the long-term effects of YoGI. Future qualitative longitudinal studies should consequently include follow-up periods of up to 3 or 12 months to capture the long-term effects. The single-center design prevents generalizability of applicability to other inpatient and outpatient settings. In future stages of this and other studies, multicenter designs should be considered.

## 5. Conclusion

The current study is the first longitudinal qualitative study on the experiences of in-patients with SSD with YoGI in a German university hospital setting. This study design was chosen to evaluate the effects of such a novel intervention, allowing for an in-depth exploration of participants’ experiences over time, thus enabling the investigation of mechanisms of action and processes. At baseline, this study identified motivators as well as psychosocial barriers that influenced the engagement in yoga practice. Four mechanisms of change were identified: acquiring competence in relaxation, increased interoceptive awareness, feeling connected, and a sense of spiritual wellbeing. While most participants experienced these positive changes, three reported adversities during YoGI, such as increased symptoms (e.g., ruminative thoughts and de-realization). To summarize, results indicate that YoGI–a low-threshold and low-cost group intervention of 4 weeks–led to increased wellbeing and substantial symptom improvements in study participants. The destigmatizing and normalizing approach of the intervention, which highlights inclusivity while tailoring the practice specifically to the needs of the targeted group, may have contributed to the improvements by reducing feelings of psychological stress, isolation, shame, and stigmatization. However, future studies are needed to verify this hypothesis.

## Data availability statement

The raw data supporting the conclusions of this article will be made available by the authors, without undue reservation.

## Ethics statement

The studies involving human participants were reviewed and approved by ethics committee of the Charité – Universitätsmedizin Berlin. The patients/participants provided their written informed consent to participate in this study.

## Author contributions

LT designed and executed the study, conducted the interviews and data analyses, and wrote the manuscript. IH and NB collaborated with the design and execution of the study and editing of the manuscript. LF assisted with the study design, supervised the data analyses, and edited the manuscript. TS assisted with conducting the data analyses and edited the final manuscript. MZ conducted the rater-blinded clinical interviews (PANSS, CDSS, and PSP) and edited the final manuscript. EH assisted with the editing of the final manuscript. KB designed the study, edited the manuscript, and supervised the study process. All authors contributed to the article and approved the submitted version.
